# Species coexistence and the superior ability of an invasive species to exploit a facilitation cascade habitat

**DOI:** 10.7717/peerj.2848

**Published:** 2017-02-21

**Authors:** Andrew H. Altieri, Andrew D. Irving

**Affiliations:** 1 Smithsonian Tropical Research Institute, Balboa, Ancon, Republic of Panama; 2 School of Medical and Applied Sciences, Central Queensland University, Rockhampton, QLD, Australia

**Keywords:** Biotic acceptance, Foundation species, Biodiversity, Niche, Invasive species, Facilitation, Nursery habitat, Positive interactions

## Abstract

Facilitation cascades generated by co-occurring foundation species can enhance the abundance and diversity of associated organisms. However, it remains poorly understood how differences among native and invasive species in their ability to exploit these positive interactions contribute to emergent patterns of community structure and biotic acceptance. On intertidal shorelines in New England, we examined the patterns of coexistence between the native mud crabs and the invasive Asian shore crab in and out of a facilitation cascade habitat generated by mid intertidal cordgrass and ribbed mussels. These crab species co-occurred in low intertidal cobbles adjacent to the cordgrass–mussel beds, despite experimental findings that the dominant mud crabs can kill and displace Asian shore crabs and thereby limit their successful recruitment to their shared habitat. A difference between the native and invasive species in their utilization of the facilitation cascade likely contributes to this pattern. Only the Asian shore crabs inhabit the cordgrass–mussel beds, despite experimental evidence that both species can similarly benefit from stress amelioration in the beds. Moreover, only Asian shore crabs settle in the beds, which function as a nursery habitat free of lethal mud crabs, and where their recruitment rates are particularly high (nearly an order of magnitude higher than outside beds). Persistence of invasive adult Asian shore crabs among the dominant native mud crabs in the low cobble zone is likely enhanced by a spillover effect of the facilitation cascade in which recruitment-limited Asian shore crabs settle in the mid intertidal cordgrass–mussel beds and subsidize their vulnerable populations in the adjacent low cobble zone. This would explain why the abundances of Asian shore crabs in cobbles are doubled when adjacent to facilitation cascade habitats. The propensity for this exotic species to utilize habitats created by facilitation cascades, despite the lack of a shared evolutionary history, contributes to species coexistence and the acceptance of invasives into a diverse community.

## Introduction

Understanding the factors that promote species coexistence, and therefore enhance biodiversity, is a fundamental goal of ecology with important consequences for ecosystem functioning, community stability, and species conservation (Chesson, 2000; Turnbull et al., 2013; Wright, 2002). Species introductions provide an opportunity to examine the interactions that determine the ability of species to establish, persist, and coexist alongside other species (Sax, Stachowicz & Gaines, 2005). Recent theoretical developments predict that facilitation by native species can play an important role in the ability of an ecosystem to accommodate exotic species and thereby lead to positive relationships between native and invasive species (Bruno, Stachowicz & Bertness, 2003; Bulleri, Bruno & Benedetti-Cecchi, 2008). Foundation species, which create and modify habitats by ameliorating biotic and abiotic stresses, offer some of the best cases to test those predictions as to how facilitation can aid in the establishment of invasive species in both terrestrial and marine ecosystems (Altieri & Van De Koppel, 2013; Bulleri, 2009).

Although some habitats are created by monotypic stands of foundation species, such as hemlock forests (Ellison et al., 2005), it is increasingly recognized that biogenic habitats are typically created by multiple foundation species such as the multiple coral species that build a reef (Angelini et al., 2011). Indirect positive interactions involving two or more foundation species result in facilitation cascades (sensu Altieri, Silliman & Bertness, 2007) that can create a unique habitat. Synergisms between foundation species through a facilitation cascade can thus have important consequences for community structure (Thomsen et al., 2010) resulting in enhanced biodiversity, abundance, and ecosystem function within the habitat patches they create (Angelini et al., 2011; Bishop et al., 2012; Dijkstra, Boudreau & Dionne, 2012; Gribben et al., 2009; Hughes et al., 2014). A mechanistic understanding of how native and invasive species interact with habitats generated by facilitation cascades is needed to determine whether the positive effects of foundation species on invasive species also occur in facilitation cascades with multiple foundation species.

Our previous work on cobble beaches of New England identified a facilitation cascade in which cordgrass (*Spartina alterniflora*) ameliorates harsh thermal conditions by providing a cool and moist habitat which allows establishment of ribbed mussels (*Geukensia demissa*), and mussels in turn facilitate other organisms by providing crevice space and a stable hard substrate for settlement (Altieri, Silliman & Bertness, 2007). This cascade of positive interactions enhances both native biodiversity and the abundance of the invasive Asian shore crab (*Hemigrapsus sanguineus*) and explains their overall positive association across the landscape (Altieri et al., 2010). A recent study in the same system revealed that the positive interactions of the facilitation cascade can expand the realized niche of some species and extend their distribution across habitat borders (Crotty & Bertness, 2015).

We hypothesized that a difference between native and invasive species in their utilization of the facilitation cascade habitat would spill over by affecting their relative abundance in the adjacent habitat, and that such a difference would be a mechanism of species coexistence that could allow the emergent pattern of a positive relationship between species diversity and invasion success. We tested that hypothesis by returning to the cobble beach study system, where the facilitation cascade was first described. We focused on interactions between the invasive Asian shore crab and native mud crabs based on their potential for diet overlap, similar distribution patterns across the shoreline, and direct antagonistic interactions (Lohrer & Whitlatch, 2002; Lohrer et al., 2000b; Williams, 1984). Earlier studies established the Asian shore crab as a voracious consumer and strong interactor capable of displacing another species of invasive crab within a few years of their arrival in our study region in the mid-1990s (Lohrer & Whitlatch, 2002). However, we observed that invasive Asian shore crabs and native mud crabs coexisted on the same beaches, and sometimes under the same rocks. Although other studies have also noted that Asian shore crabs and native mud crabs have overlapping distributions, and that both have the potential to consume other species of crabs, no prior work has conducted the experiments to examine their direct interactions with one another or the relative importance of their interactions with biogenic habitats (Epifanio, 2013). Recent food web and interaction network analyses in the same intertidal system suggests that the importance of positive interactions in the form of habitat modification outweigh consumptive interactions for determining community structure and diversity patterns (van der Zee et al., 2016), and we hypothesized that the facilitation cascade played a role in the persistence of potentially interacting species. Within this model study system, we examined mechanisms of species coexistence by conducting a series of surveys and experiments to test the potential and realized use of the cordgrass–mussel facilitation cascade habitat and adjacent cobble habitat by invasive Asian shore crabs and native mud crabs, as well the direct interactions of the crabs with one another.

## Materials and Methods

### Study system

We examined the role of facilitation cascades and secondary species interactions in species coexistence on cobble beaches in Narragansett Bay, Rhode Island where we first described facilitation cascades and the positive relationship between native biodiversity and invasion success (Altieri, Silliman & Bertness, 2007; Altieri et al., 2010). We conducted field surveys in Brown University’s Haffenreffer Reserve (41°41′N, 71°14′W), Colt State Park (41°41′N, 71°18′W), and the Narragansett Bay National Estuarine Research Reserve (NB-NERR) on Prudence (41°39′N, 71°21′W) and Patience (41°39′N, 71°22′W) Islands and experiments within the NB-NERR. Cordgrass (*S. alterniflora*) and ribbed mussels (*G. demissa*) form discrete beds, hereafter “cordgrass–mussel beds,” at mid intertidal heights that are interspersed with areas of bare, unconsolidated cobblestones, hereafter “mid cobble.” Cordgrass–mussel beds are confined to the mid-intertidal, and below this zone the low intertidal is comprised entirely of cobblestones, hereafter “low cobble.” We focused on coexistence between the invasive Asian shore crab (*H. sanguineus*) and native mud crabs (Panopeid crabs that were predominantly *Eurypanopeus depressus* with occasional *Dyspanopeus sayi*). Asian shore crabs became established in our study region by 1996, and within three years they are thought to have caused green crabs (*Carcinus maenas*) to become rare or absent on cobble shorelines (Lohrer & Whitlatch, 2002). Scientific collecting permits were granted by Rhode Island Department of Environmental Management (permits #2007, 2008–47).

### Surveys of habitat use and distributional overlap of crabs

To quantify whether there was evidence for spillover effects adjacent to cordgrass–mussel beds, we conducted a survey in August 2007 to compare the abundance of adult crabs in low cobble habitats at sites with cordgrass-ribbed mussel beds in the mid zone vs. sites without beds in the mid zone. We counted and measured all crabs within eight 25 cm × 25 cm quadrats in the low cobble zone at 14 sites: seven replicate sites where the low cobble was below and adjacent to cordgrass-ribbed mussel beds (< 5 m distance) and seven replicate sites where the low cobble was not adjacent to a bed (> 200 m distance from sampling area to nearest beds). The average number of crabs per quadrat at each site was used as the response for each replicate site, and the difference between sites with and without cordgrass-ribbed mussel beds in adult crab densities was analyzed for each species using a one-way ANOVA. This and all other statistical analyses were performed with JMP (v11; SAS Institute, Cary, NC, USA).

We conducted a second survey in September 2007 to examine patterns of coexistence between Asian shore crabs and mud crabs, and their relative abundance in different habitat types. We selected 12 replicate sites that each had all three habitat types present (low cobble, mid cobble, and cordgrass–mussel bed), and then quantified the abundance, identity, and size of all crabs in eight 25 cm × 25 cm quadrats in each habitat type at each site. For life history stage, mud crabs and Asian shore crabs were considered recruits at carapace width ≤ 8 and ≤ 10 mm, respectively, which were the smallest sizes of individuals that were gravid and sex was distinguishable based on abdomen morphology, and largest sizes of individuals that recruited into our crab interaction field experiment. The average number of crabs per quadrat in each habitat was used as the response for each replicate site, and differences between habitats were analyzed for each species and life history stage with a nested ANOVA, followed by Tukey’s HSD post hoc analysis, to determine which habitats differed for each species and stage combination. Data were log transformed to meet assumptions of the analysis. To analyze the small-scale relationships between the abundance of Asian shore crabs and mud crabs, we used a Pearson’s correlation analysis of the average number of each species found in the low zone quadrats at each site as the response variable. We also explored whether the Asian shore crabs are recruitment-limited by testing for a correlation across sites between recruit and adult densities (since recruits and adults tended to be found in different habitats within a given site, we averaged all quadrats from all habitat for each life history stage at each site). Larval duration for Asian shore crabs is ∼2 to 3 weeks (Epifanio, 2013), and transport of larvae is highly dynamic since the residence time of Narragansett Bay waters can vary from 2–5 weeks and strongly influences larval retention within the bay (Gaines & Bertness, 1992).

### Settlement experiment

We measured crab settlement patterns across all habitat types by deploying standardized settlement modules in each of the three habitat types (mid cobble, low cobble, and cordgrass–mussel bed) at each of four sites for six weeks ending in October 2008 (*n* = 5 replicate modules per habitat per site, > 5 m between modules). Modules were comprised of plastic tubs (10 cm length × 10 cm width × 12 cm height) filled with beach material from the study area that has been rinsed and cleared of any visible live organisms. The modules were covered with plastic mesh (7 mm mesh size) to minimize predation on recruits within, had 1 mm diameter holes drilled in their bottoms to allow drainage, and were secured to stakes with their lip 6 cm above the surrounding substrate surface to minimize the likelihood of crab recruits from walking into the modules from the surrounding habitat. At the end of the experiment, the modules were carefully placed in bags to prevent loss of potentially fleeing crabs and then transported to the laboratory where the contents of each bags were examined and all crabs identified and counted. Differences across habitat types in abundance of recruits for each species were examined with a nested ANOVA with site nested within habitat type.

### Transplant experiment

To test whether Asian shore crabs and mud crabs could similarly benefit from habitat amelioration by the cordgrass-ribbed mussel facilitation cascade, we transplanted both crab species into cordgrass–mussel beds, mid cobble, and low cobble zones in July 2008. Thirty adult crabs of each species were secured within each habitat by 10 cm tethers, and half of the crabs in each habitat were enclosed in a cylindrical cage (30 cm diameter × 20 cm height, galvanized steel mesh with 7 mm opening size) to test for predation pressure and tether failure (*n* = 180 crabs total). Crabs were scored for survivorship after 24 h, and the proportion of each species surviving in each habitat were analyzed with a chi-squared test. Preliminary analysis revealed no difference in survivorship between caged and uncaged individuals for all zones and species (indicating no effect of predation or tether failure), so caged and uncaged treatments were pooled for each species and zone combination.

### Crab interaction experiments

We conducted field and laboratory experiments to test for negative interactions between Asian shore crabs and mud crabs. The first experiment conducted in the field used mesh enclosures in the low cobble to test the effect of adult crabs of each species on patterns of crab recruitment as well as survivorship of adults of the other species. Mesh enclosures were set > 2 m apart on a cobble beach, with their roofs set flush with the surrounding substrate, and were filled with beach material (from which crabs were removed) collected from the local beach area. Enclosures were then stocked with adult crabs to achieve six treatments (*n* = 12 per treatment): no crabs, two Asian shore crabs, two mud crabs, four Asian shore crabs, four mud crabs, and a mix of two Asian shore crabs plus two mud crabs (stocking densities were within natural densities observed in our surveys). The four-crab monospecific treatments were included to test whether survivorship differences between the two-crab monospecific treatments and four-crab mixed species treatments were due to inclusion of a second species per se, or simply higher crab density overall. Since initial densities of a given species were double in four-crab monospecific treatments relative to the other treatments, their final densities were halved for analysis to allow standardize per capita survivorship values across all treatments. We tested the generality of interactions by including crabs of varying sizes in the experiment (carapace width range of 12–23 mm), however, crabs used in any particular replicate were matched to have similar carapace width (within 3 mm). Enclosures were 30 cm length × 30 cm width × 15 cm height with four walls, floor, and roof constructed of plastic mesh (7 mm mesh size). The experiment ran for two months from June to August 2007, and at the end of experiment we counted the final number of surviving adults and new recruits of each species in each enclosure. Some replicates were excluded from analyses because their portion of the beach unexpectedly became inundated by fine sediment, leaving ≥ 8 replicates per treatment. To examine the effect of interspecific interactions among adults, we conducted a one-way ANOVA on final abundance of adults of each species that included three treatment levels: two-crab monospecific and four-crab monospecific treatment for the species of interest, and four-crab mixed treatments that also included the other species. To examine the effect of adult crabs on recruitment, we conducted a two-way ANOVA on the final abundance of recruits of each species by crossing the presence or absence of two adult mud crabs with the presence or absence of two adult Asian shore crabs.

Since our field experiment indicated that recruitment of Asian shore crabs was lower in enclosures that contained adult mud crabs, we conducted a second experiment in August 2008 that examined their relationship in greater detail by measuring survivorship when they were placed alone or together in laboratory arenas. The arenas were comprised of opaque plastic cylinders (18 cm diameter × 13 cm height) filled to a depth of 4 cm with seawater, and each contained a single shelter (5 cm length × 2.5 cm width × 1.2 cm height). Arenas were kept on a day/night light cycle of 14 h light/10 h dark. The shelter provided a resource to examine potential competitive interactions since both species showed a strong preference for the shelter when in the arena alone (≥ 75% occupancy), and we never observed more than one crab in the shelter when two crabs were in the arena. We established 12 replicate arenas for each of three treatments: mud crab adult alone, Asian shore crab recruit alone, and mud crab adult and Asian shore crab recruit together. The size range of mud crabs adults and Asian shore crabs recruits were 14–20 and 7–9 mm, respectively. After 48 h, we scored each crab for survivorship (alive or dead) and occupancy (inside or outside of shelter). Differences in Asian shore crab recruit survivorship between treatments with and without adult mud crabs were analyzed with chi-squared analysis.

## Results

### Surveys of habitat use and distributional overlap of crabs

**Figure 1:**
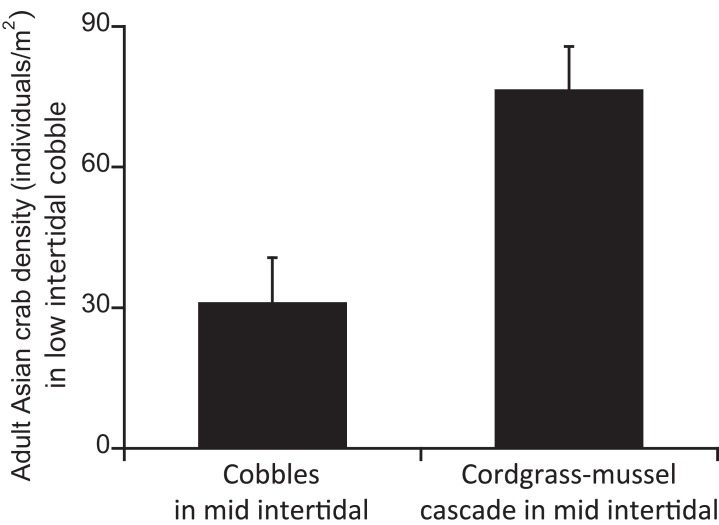
Densities of Asian shore crabs in low cobble area with and without adjacent cordgrass–mussel beds in the mid intertidal. Data are mean + SE.

Our first survey of adult crab densities revealed that densities of Asian shore crabs in the low cobble zone were greater at sites with adjacent cordgrass-ribbed mussel beds in the mid zone than at sites without beds (*F*_1,12_ = 11.80, *P* < 0.001; Fig. 1), whereas mud crabs showed no difference between sites with and without adjacent beds (*F*_1,12_ = 4.14, *P* > 0.05).

**Figure 2:**
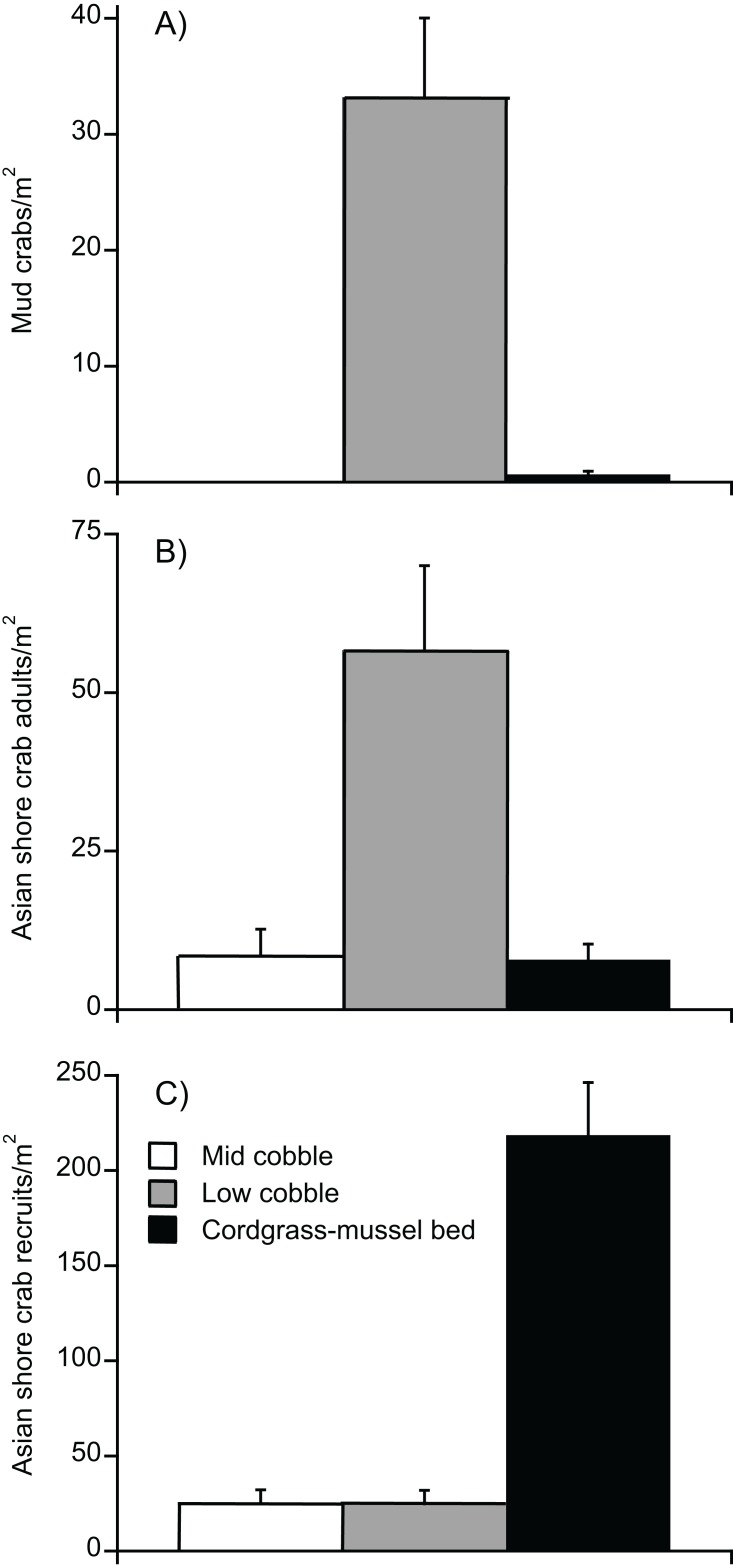
Distribution of crabs across intertidal landscape. (A) Mud crabs (with the exception of six individuals) were found exclusively in the low cobble zone (adults and recruits pooled together in figure). (B) Adult Asian shore crabs followed a similar pattern to mud crabs in that they were found predominantly in the lower cobble zone. (C) Recruits of the Asian shore crabs were found mostly within the cordgrass–mussel habitat created by the facilitation cascade. Data are mean + SE.

In our second survey of crab distributions, we found that all species and stages differed by habitat type (*F*_2,33_ > 16.04, *P* < 0.0001 all analyses). Tukey’s HSD post hoc analysis revealed that densities of mud crab adults and recruits, as well as Asian shore crab adults, were highest in the low cobble zone with no difference between mid cobble and cordgrass–mussel beds, whereas Asian shore crab recruits were highest in the cordgrass–mussel beds with no difference between mid and low cobble zones (Fig. 2). A total of only six mud crabs were found anywhere in the mid zone, of which four were within the cordgrass-ribbed mussel bed (and all six were recruits).

The density of adult Asian shore crabs was independent of adult mud crabs in the low intertidal (*R*^2^ = 0.22, *P* = 0.13), indicating that the two species coexist.

There was a positive correlation between densities of recruit and adult Asian shore crabs across the 12 study sites (*R*^2^ = 0.44, *P* < 0.05), suggesting that their populations are recruitment-limited.

### Settlement experiment

**Figure 3:**
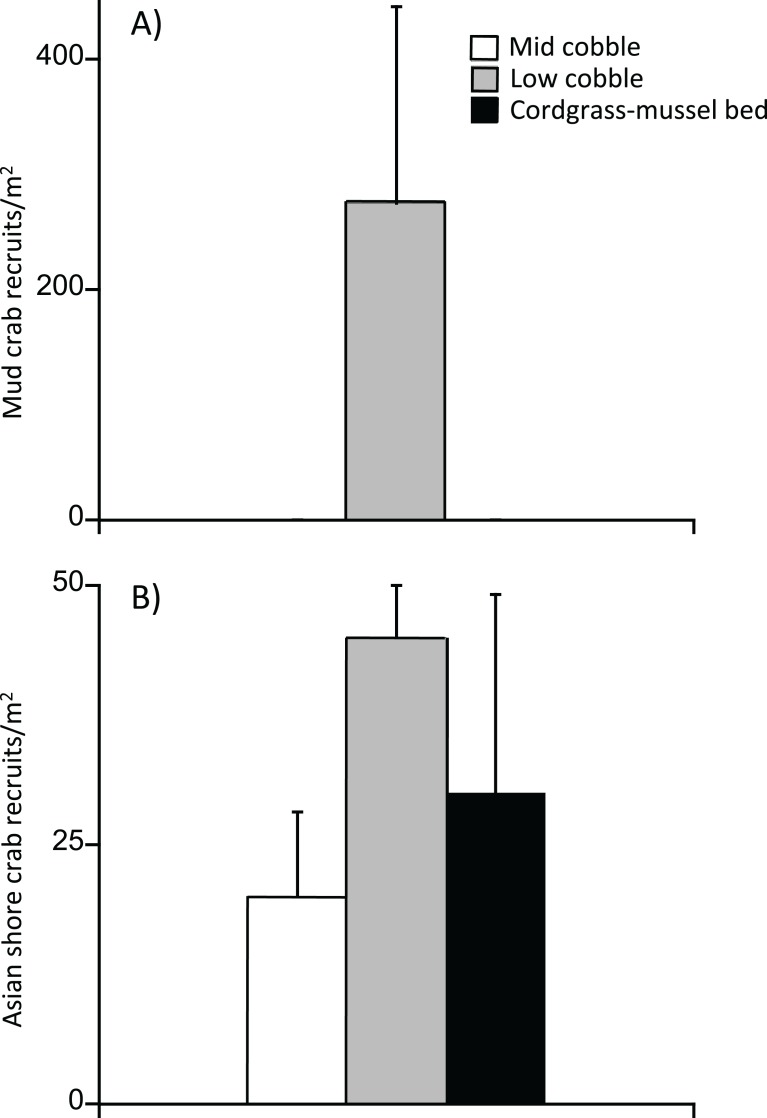
Settlement rates of (A) native mud crabs and (B) invasive Asians shore crabs into mid cobble, low cobble, and cordgrass–mussel bed zones. Data are means + SE.

Standardized substrate modules revealed differences between the two crab species in their settlement patterns across the intertidal landscape (Fig. 3). Settlement rates of Asian shore crabs were similar across mid cobble, low cobble, and cordgrass-ribbed mussel bed habitats (*F*_2,48_ = 0.92, *P* = 0.41). Settlement of mud crabs differed by habitat (*F*_2,48_ = 24.30, *P* < 0.0001), and they settled exclusively in the low cobble zone.

### Transplant experiment

**Figure 4:**
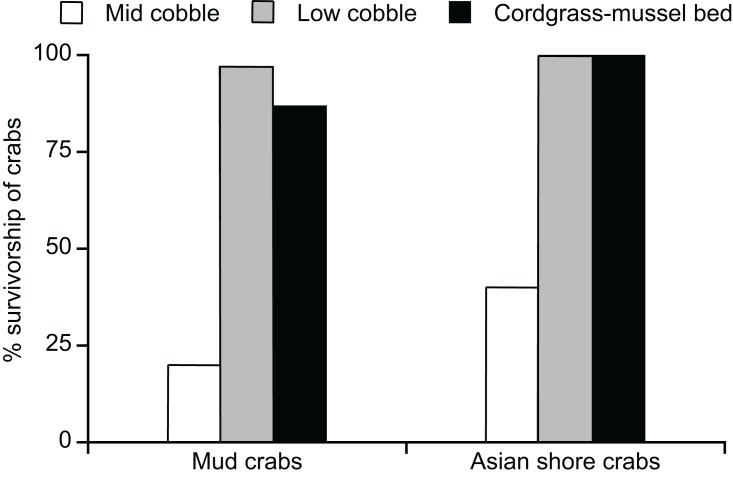
Survivorship in transplant experiment. Both crabs benefited from the facilitation cascade which resulted in survivorship that was higher in the cordgrass-mussel bed than mid cobble at the same tidal height, and similar to that found in low cobbles.

Asian shore crabs had 100% survivorship in low cobble and cordgrass-ribbed mussel beds, which was greater than their survivorship in mid cobble habitat (*χ*^2^_1,88_ = 49.69, *P* < 0.0001) (Fig. 4). Likewise, mud crabs had similarly high survivorship in low cobble and cordgrass-ribbed mussel beds (*χ*^2^_1,58_ = 0.15, *P* < 0.1482) and lower survivorship in the mid cobble (*χ*^2^_1,88_ = 48.692, *P* < 0.0001).

### Crab interaction experiments

**Figure 5:**
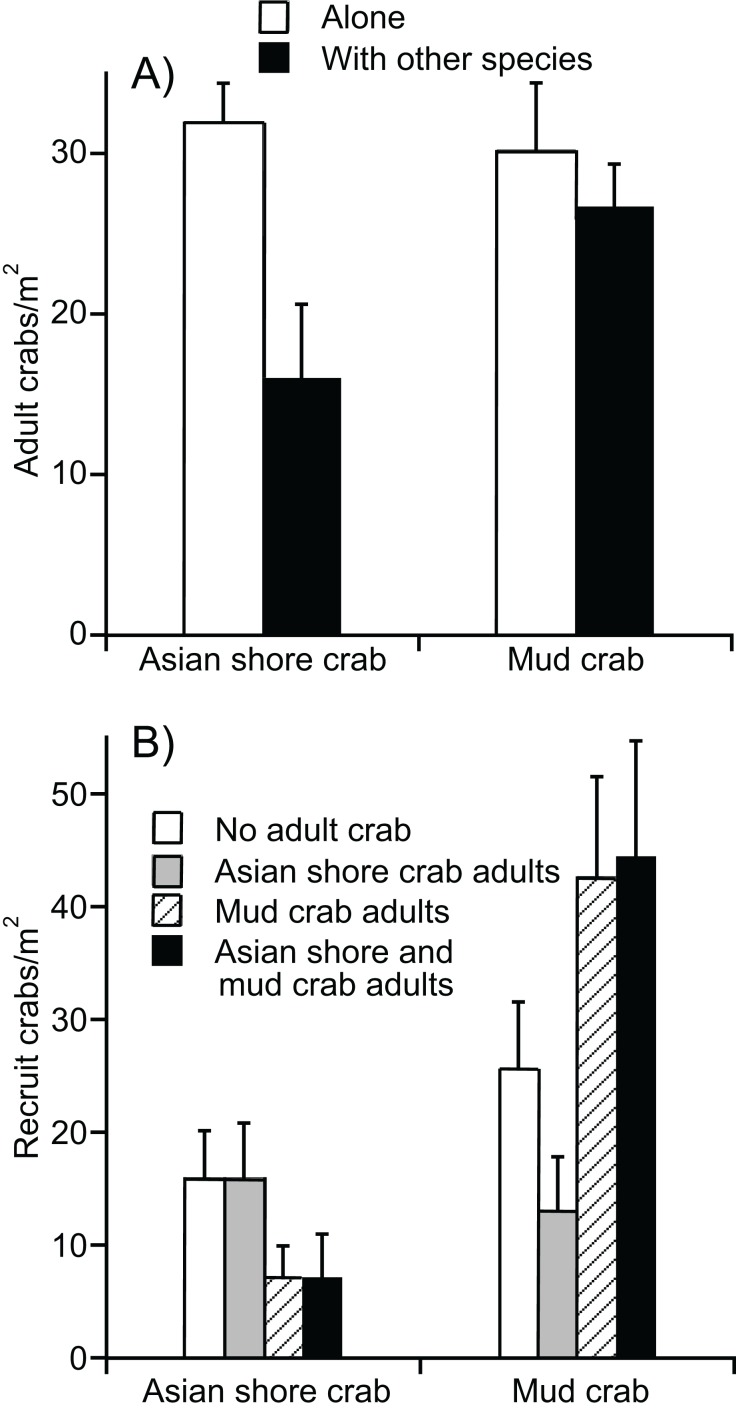
Experimental evidence for mud crab resistance to Asian shore crabs. (A) Densities of adults remaining in enclosures as a function of initial crab addition treatments. (B) Densities of recruits in enclosures as a function of initial crab addition treatments. Data are mean + SE.

Our field enclosure revealed a unidirectional antagonistic relationship in which adult mud crabs had a negative effect on the density of adult Asian shore crabs (*F*_2,24_ = 6.53, *P* < 0.01). The Tukey’s HSD post hoc revealed that the Asian shore crab density was lower when mud crabs were added to enclosure, but not when other Asian shore crabs were added. Asian shore crabs had no effect on densities of adult mud crabs (*F*_2,25_ = 6.60, *P* < 0.01). The corresponding Tukey’s HSD post hoc revealed no effect on adult mud crab density when Asian shore crabs were added; however, adding other mud crabs decreased survivorship suggesting some self-thinning (Fig. 5A).

We also found a negative effect of adult mud crabs on densities of Asian shore crab recruits in the enclosures (*F*_1,35_ = 4.37, *P* < 0.05) (Fig. 5B). In contrast, mud crab adults had a positive effect on densities of mud crab recruits (*F*_1,35_ = 9.97, *P* < 0.05). There was no significant effect of Asian shore crab adults on recruitment of Asian shore crabs or mud crabs (*F*_1,35_ < 1.30, *P* > 0.25 both analyses).

Our laboratory arena experiment revealed strong negative effects of mud crab adults on survivorship of Asian shore crab recruits (χ^2^_1,22_ = 15.28, *P* < 0.0001). Asian shore crab recruits had 100% survivorship and 100% shelter occupancy when alone in the arena, however, their survivorship dropped to just 25% in the presence of adult mud crabs. Those Asian shore crab recruits remaining alive were always displaced from the shelters, as they were only observed in the open whereas mud crabs were always found to occupy the shelter when the two were together.

## Discussion

**Figure 6:**
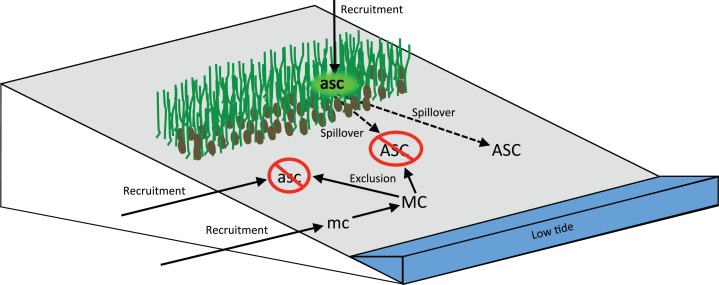
Diagram representing how the cordgrass–mussel facilitation cascade promotes species coexistence on intertidal cobble beaches. The dominant mud crab reduces the abundance and survivorship of the invasive Asian shore crab in the lower intertidal cobble zone. The abundance of Asian shore crabs in the low intertidal, where their distribution overlaps with mud crabs, is more than double adjacent to cordgrass–mussel beds relative to stretches of beach without the cordgrass–mussel facilitation cascade. This suggests that spillover (dashed arrows) of Asian shore crabs from cordgrass–mussel bed nursery habitat into the lower intertidal likely counter the deleterious effects of mud crabs. ASC, Asian shore crab adults; asc, Asian shore crab recruits; MC, mud crab adults; mc, mud crab recruits; green lines with brown ovals, cordgrass with mussels comprising the mid-intertidal facilitation cascade habitat.

We found that native mud crabs are dominant to invasive Asian shore crabs at two life history stages by negatively affecting both adult survivorship and recruitment. However, the two species coexist, and their differential use of the habitat created by the cordgrass–mussel facilitation cascade likely contributes to this pattern. Both species can benefit from habitat amelioration of the cascade, but only the Asian shore crab settles into that cordgrass-ribbed mussel habitat which serves as a nursery habitat free of the predatory mud crabs. The Asian shore crabs are recruitment-limited, and their recruitment rates are nearly an order of magnitude higher inside the cordgrass-mussel beds. The density of Asian shore crab adults is higher when adjacent to the cascade-generated habitat, suggesting that spillover from that nursery habitat subsidizes adult populations and contributes to their persistence in the low cobbles despite the presence of the dominant mud crabs (Fig. 6).

The distribution of crabs and their settlement rates indicates very different patterns of how the two species interact with the facilitation cascade. Our settlement modules revealed that Asian shore crabs arrive at similar rates in all three habitats we examined, but the greatest number of recruits were found within the cordgrass–mussel bed habitat. The relatively higher densities in the beds could be explained by mortality in mid cobble habitat due to physical stress, as found in the transplant experiment, and direct consumption and/or risk of consumption by mud crabs in the low cobble zone, as found in the field enclosure and laboratory arena. Previous experimental evidence revealed that the high abundance of Asian shore crabs within the beds is a product of the facilitation cascade since removal of one or both foundation species resulted in reductions or loss of the crabs (Altieri et al., 2010). Unlike the recruits of Asian shore crabs found largely in the beds, we found that Asian shore crab adults were found primarily in the low cobble. Mud crabs do not show any association with the facilitation cascade as they were confined to the low cobble zone as both adults and recruits, and their densities are not affected by whether or not a cordgrass–mussel bed is in the adjacent mid intertidal. Thus, mud crabs do not take advantage of the shelter of the cordgrass–mussel bed habitat despite evidence from the transplant experiment that they can benefit from stress amelioration of the cordgrass–mussel bed habitat. As a consequence, the cordgrass–mussel beds are relatively free of mud crabs, and are therefore a habitat with reduced mortality risk and enhanced recruitment for Asian shore crabs.

Why do Asian shore crabs and mud crabs differ in their utilization of the facilitation cascade-generated habitat? The Asian shore crab is a shelter dependent species that preferentially associates with increased habitat complexity on cobble beaches in its native range (Lohrer et al., 2000a), and they will occupy marshes when cobbles or burrow structures are coupled with vegetation (Brousseau, Kriksciun & Baglivo, 2003; Lohrer et al., 2000b). These habitat associations predict the high abundance of early life history stage Asian shore crabs in the cordgrass–mussel bed habitat. Since our transplant experiment revealed that mud crabs can also benefit from association with the cordgrass–mussel beds, as found using short-term transplants of many other species in the same system (Crotty & Bertness, 2015), some other factor such as inundation time, food availability, or substrate suitability for burrowing appears to be dictating their fidelity for soft sediments underlying the low cobble zone rather than the mussels and well-drained cordgrass peat in beds. Thus, the invasive Asian shore crabs, which have been in this ecosystem for only a few decades, have a superior ability to exploit the beds relative to the native mud crabs, which have a shared evolutionary history with the cordgrass and mussels. The paucity of native mud crabs in the cordgrass–mussel beds suggest that the beds essentially provide a different and unutilized niche which is thought to increase the likelihood of invasion (Brown, 1989; Davis, Grime & Thompson, 2000). Whether invasive species occupy unused niches or displace native species from an occupied niche is a fundamental question in invasion biology (Vermeij, 1996), and on cobble beaches the former appears to be the case for shore crabs. Differences such as those we identified between adjacent habitats in their suitability for the native and invasive species can be important for management of biodiversity and conservation (Fischer et al., 2009).

Consumption by native species is thought to be an important mechanism of biotic resistance that is widespread across terrestrial and aquatic ecosystems (Kimbro, Cheng & Grosholz, 2013; Levine, Adler & Yelenik, 2004; Parker, Burkepile & Hay, 2006). We found that predation by native mud crabs can reduce recruitment rates of invasive Asian shore crabs, indicating that some measure of biotic resistance is operating in our study system. However, it has evidently been insufficient to repel invasion by Asian shore crabs. We hypothesize that the cordgrass–mussel beds promotes this coexistence between crabs in the adjacent low cobble zone by providing a refuge where Asian shore crabs can mature to a size where they are more resistant to predation by mud crabs, and/or by sufficiently enhancing recruitment rates such that the number of Asian shore crabs generated by the cordgrass–mussel beds can overcome predation rates. The lack of predatory mud crabs in the cordgrass–mussel beds supports the former refuge hypothesis, whereas the findings that Asian shore crabs in cordgrass–mussel beds reach some of the highest densities worldwide including in their native range (Altieri et al., 2010), and the overall higher density of Asian shore crabs than mud crabs in the lower cobble zone, supports the latter predator-swamping hypothesis. The complexity of the cobble habitat may also contribute to persistence of the two crab species within the same zone (Langellotto & Denno, 2004).

We found evidence for spillover effects of facilitation cascades. The cordgrass–mussel beds functioned as a nursery habitat for Asian shore crabs which was free of predatory mud crabs, and which had recruitment rates nearly an order of magnitude higher than areas outside beds. Asian shore crabs likely move from the beds into immediately adjacent cobble areas which are well within the 5 m daily range of movement Asian shore crabs (Brousseau et al., 2002). This would explain why densities of adults in low cobble habitat were more than doubled when adjacent to the beds. It is increasingly recognized that biodiversity and dynamics in a given habitat are dependent on other habitats in the surrounding landscape (Saunders, Hobbs & Margules, 1991; Turner & Garder, 2015), as appears to occur in our study system where the association between native and invasive species in the cobble habitat is apparently influenced by the facilitation cascade in the adjacent cordgrass–mussel habitat. The interaction between habitats that occurs through the movement of materials, trophic resources, propagules, and foraging organisms has been variously studied as “edge effects” (Leopold, 1933), “adjacency effects” (Hersperger, 2006; Hersperger & Forman, 2003), and “spillover effects” (Gell & Roberts, 2003) that are more broadly part of a suite of long distance interactions that structure communities and link ecosystems (van de Koppel et al., 2015).

While we detected spillover of Asian shore crabs from the cordgrass–mussel bed to the immediately adjacent cobble habitat, the crab populations at sites without beds may have been subsidized through migration of adults and/or transport of larvae from abundant populations associated with distant beds within the bay. The contrast between sites with and without beds, and therefore the apparent contribution of spillover from the facilitation cascade habitat, may have been greater without such subsidies which were likely enhanced in our semi-enclosed estuary system that has the potential for significant larval retention and demographic linkages (Gaines & Bertness, 1992). Further work at regional and global scales would determine the potential for such spillover from facilitation cascade habitats to occur at larger spatial scales and to promote range expansion of invasive species.

In addition to the greater recruitment of Asian shore crabs to the cordgrass–mussel beds, and the related spillover from this facilitation cascade habitat to the lower intertidal, there are several other potential complementary hypotheses to explain the pattern of greater Asian shore crab abundance in the low cobble zone when adjacent to the beds. First, greater delivery of propagules may have contributed to the co-occurrence of higher abundances Asian shore crabs and the establishment of cordgrass–mussel beds in high-delivery areas. Prior work in this system suggests this explanation is unlikely since passive delivery of plant seeds was estimated to be similar to beach areas with and without beds (Bruno, 2000). Moreover, water movement, which is a proxy for the water flow that would deliver propagules, does not differ between stretches of cobble beach with and without cordgrass–mussel beds (van Wesenbeeck et al., 2007). Second, greater food availability associated with the high productivity of cordgrass–mussel beds, which have a higher abundance of invertebrate and algal resources than bare cobbles at the same height (Altieri, Silliman & Bertness, 2007), may have enhanced adjacent population densities of Asian shore crabs through increased settlement or post-settlement survivorship. Our settlement experiment failed to find habitat-specific settlement patterns, which suggests that crab settlement was independent of any potential variation in food availability among habitats as found for other crab species in this system (Lindsey, Altieri & Witman, 2006). It is also unlikely that post-settlement survivorship was enhanced by potentially higher food availability in beds since most large Asian shore crabs were found in the low cobble zone rather than foraging in the cordgrass–mussel beds, and the low cobble zone has densities of many prey items (e.g., barnacles and blue mussels) that rival those found in the beds (Altieri, Silliman & Bertness, 2007). Third, food resources associated with cordgrass–mussel beds could have supplemented the diet of mud crabs and relieved predation pressure on Asian shore crabs. In addition to the apparent lack of food limitation in the low intertidal as described above, this explanation seems unlikely because our experiment which found mud crabs could limit Asian shore crab recruitment was conducted in the area adjacent to cordgrass–mussel beds, and because if any supplemental food resources did increase the abundance of mud crabs, then there could be a negative effect on Asian shore crabs through apparent competition (Holt, 1977).

It has been recognized that general patterns of biodiversity are the net product of positive and negative interactions (Bruno, Stachowicz & Bertness, 2003). Our study suggests likewise, where the success of invasive Asian shore crabs, and coexistence of a native and invasive species, is a consequence of the interplay between negative (i.e., interaction dominance of mud crabs) and positive interactions (i.e., habitat creation by the cordgrass–mussel facilitation cascade). We suggest that predicting which introduced species have the potential to be invasive, and which ecosystems are most likely to be invaded, needs to consider not only the negative and positive species interactions, but also their potential interplay with habitats that can generate spillover effects across landscapes. Moreover, outcomes may not be predictable based on how native species utilize habitat as we found an apparently underutilized niche for invasive crab occupancy and settlement in the beds formed by the facilitation cascade.

## Supplemental Information

10.7717/peerj.2848/supp-1 Supplemental Information 1 Raw data.

## Additional Information and Declarations

### Field Study Permissions

The following information was supplied relating to field study approvals (i.e., approving body and any reference numbers): Rhode Island Department of Environmental Management (permits# 2007, 2008–47).

### Data Deposition

The following information was supplied regarding data availability: The raw data has been supplied as Supplemental Dataset Files.
